# Tennessee State Medical Society

**Published:** 1891-06

**Authors:** 


### TENNESSEE STATE MEDICAL SOCIETY.

FIFTY-EIGHTH ANNUAL MEETING HELD IN NASHVILLE, APRIL 14TH, 15TH, AND 16TH, 1891.

### SECOND DAY—*Morning Session.*

[Concluded from Page 247.]

DR. J. S. CAIN, of Nashville, read a paper on CHRONIC ENDOMETRITIS.

He said the question as to the localization of the chronic form of endometritis is rendered more prominent than that of the acute form on account of the conflicting opinions entertained by distinguished authors and teachers. None, he believed, questioned the very frequent occurence of chronic cervical endometritis, but Drs. Emmett, Bennett, and other very distinguished authorities, almost absolutely ignored the existence of chronic corporal endometritis as a special disease, and conseqently except for the relief of hemorrhages, and to meet temporary emergencies, discountenance all intra-uterine modification, relying entirely upon treatment directed to the uterine os and vault of the vigina. But the great preponderance of medical authority is averse to the opinions entertained by these gentlemen, and with the latter class he was entirely in accord.

Chronic endometritis and the conditions necessarily allied therewith are the most common as well as the most important diseases with which the gynaecologist has to deal. This condition is often a sequel to the acute form of the disease, and grows out of repeated acute attacks. It matters not how or from what source the acute outbreaks originate, whether from catarrhal, specific, traumatic or internal constitutional causes, they are often, but not always, the starting point from which not only the endometrium but the entire uterine and periuterine parenchymatous structures become involved. He would here venture the assertion, that while the change in structure and function of the lining membrane of the uterus often seem to be the most prominent conditions, and those which demand our first and most careful attention, this tissue is probably never chronically diseased without a corresponding involvement of the entire uterine structures.

*Treatment*—While the curette, as has been said, is a blind instrument and capable of doing much harm in careless incompetent hands, yet for the removal of fungoid vegetations and adenoid degenerations from the endometrium, it affords the surest, speediest and safest means as yet devised. Dr. Cain is accustomed to follow the curetting by an application of Churchill's tincture of iodine or diluted carbolic acid,as is the usual practice, and always precedes the treatment by a careful washing out of the vagina and uterus with a disinfectant of one or two thousand corrosive sublimate.

In cases where this treatment is not admissible, or where it has failed to afford relief, his next reliance is on the electro-chemical action of negative galvanism, in removing the vegetations after the method of Apostoli. This is accomplished by introducing an electrode, insulated to near the point into the uterine cavity, and connecting with the negative pole of the battery, connecting the other pole with a large pad of moistened potter's clay, sponge or prepared cotton, placed over the abdomen. The time for the employment of the galvinism at each teatment should be from ten to fifteen minutes, and the treatment should be repeated about twice a week.

The strength of the current employed will depend much upon the acuteness of the particular case, and the susceptibility of the patient to electrical treatment. The chronic cases always require the stronger current. The dosage may be fixed at from ten to three huudred milliamperes; the minimum is, in his judgment, too small to accomplish any results, and yet physicians with much larger experience than himself, have had to employ it.

This line of treatment he considers free from many of the objections to others; it is cleanly, free from pain, and exempt from danger. Unlike cauteries and escharotics it can be limited in its influence and produces no deleterious effects upon the sound tissues, nor does it leave a raw and exposed surface like the curette to absorb poisons and septic agencies, and while it removes the vegetations it imparts renewed tone and vitality to the diseased organ.

---

SECOND DAY—*Afternoon Session.*

DR. A. J. SWANEY, of Gallatin, contributed a paper entitled Retained Placenta in Miscarriage ; How shall We Treat Such Cases. (Which will appear later.)

DR. J. L. JONES, of Bells, read a paper on Indigo as an Emmenagogue, in which he said his attention was first directed to this drug as an emmengogue in July, 1887, from an essay published in the Medical and Surgical Reporter, of Philpdelphia, by Dr. S. L. Gount, of Lafayette, Ind. Acting on the suggestions offered by Dr. Gount he had used it in many and various cases.

His first case was a young lady, twenty years of age, who had not menstruated in five months. He had been treating her for three months with the usual remedies without any effect, so made up his mind to give indigo a trial, which he did with the following result :

He ordered indigo, ℥ ij, subnitrate of bismuth, ℥ ss, well mixed. She took one-half teaspoonful in one-third of a glass of water three times daily for nearly four weeks, when one day he was sent for in great haste to see his patient. On his arri val he found her on the bed and comfortable. Having asked why he was so hastily called, he was told by the mother that her daughter while walking in the garden, without any pain or warning of any kind, she began to flood. The gush was followed by a gentle flow which lasted only for a little while. In five days she was well, and has not suffered from amenorrhœa since.

Dr. Jones has since used indigo in thirteen cases with but one failure, and that lady proved to be pregnant.

During the administration of the drug the os uteri becomes soft and patulous, admitting the end of the index finger. There is often a serous discharge from the vagina. The urine becomes brownish-green in color, and its odor is offensive. The stools are watery and offensive.

---

THIRD DAY—*Morning Session.*

DR. J. A. WITHERSPOON, of Columbia, read a paper on Diabetes. (Which will appear later.)

TREATMENT OF STRICTURES OF THE MALE URETHRA.

This was the title of a paper read by Dr. J. W. Handly, of Nashville.

He said strictures of large calibre, if they be recent, but poorly organized and of the linear variety, may be treated by dilatation, which must be continued for months. But should the stricture be densely fibrous, and not easily dilatable, the cutting operation becomes necessary, for which purpose he prefers Dr. Otis' improved dilating urethratome, with which the surgeon can accurately divide any stricture to any size desired.

Strictures of small calibre, situated in advance of the bulbo-membranous junction, unless seen very early and found to be unusually soft and dilatable, furnish a typical condition for internal urethrotomy, that in which it is attended with the least possible danger and greatest prospect for a permanent cure. Should the contraction be so great that the Otis urethratome cannot be used, had found Bank's whalebone dilator, which are made in four sizes, of great advantage in opening the canal so as to admit of the urethratome, and considered them very useful.

Strictures of small calibre posterior to bulbo-membranous junction, require a very different course of treatment, since internal urethrotomy at this point is often attended with profuse hemorrhages, fever, rigors or other disturbances equally as disagreeable. Strictures of this variety permeable only to filiform bougies, may be treated in one of the four following ways, to-wit:

1. After the filiform has been introduced it may be allowed to remain *in situ* for two or three days and another passed alongside of it to serve as a guide for the introduction of a tunnelled sound, later to be followed by the ordinary soft or steel bougies. This is good and safe surgery in the absence of retention.

2. The surgeon may attempt to conduct a tunnelled sound over it at once, to be followed by gradual dilatation.

3. He may conduct over it a grooved staff and then proceed to the performance of external urethrotomy.

4. He may use the staff as a guide for the Massionneuve urethratome and may immediately perform internal urethrotomy.

OFFICERS FOR 1892.

The following officers were elected for the ensuing year:

President—Dr. J. W. Penn, Humbolt.

Vice President for Middle Tennessee—Dr. J. A. Witherspoon, Columbia.

Vice President for East Tennessee—Dr. C. E. Ristine, Knoxville.

Vice President for West Tennessee—Dr. C. H. Lovelace, Dukedom.

Secretary—Dr. D. E. Nelson, Chattanooga.

Treasurer—Dr. J. P. C. Walker, Dyersburg.

Place of meeting, Knoxville, second Tuesday in April, 1892.

---

SUMMER DISTURBANCES OF CHILDREN.—In fermentative disorders of the alimentary canal in the young, middle-aged or old, Listerine has given most satisfactory results. In the summer diarrhoea of children, Dr. I. N. Love, of St. Louis, speaks very highly of it, given in combination with glycerine and simple syrup. A formula that I have time and again used—in fact, it has almost become routine with me of late years—is as follows:

| | | |
|---|---|---|
| ℞ | Bismuth Sub. Nit, - - | half a drachm. |
| | Tr. Opii, - - - - | twenty drops. |
| | Syr. Ipecac, - - - | |
| | Syr. Rhei Arom, - - | aa two drachms. |
| | Listerine, - - - - | half ounce. |
| | Mist. Creta, - - - - | one ounce. |

M. Sig.—Teaspoonful as often as necessary, but not more frequently than every three or four hours. This for children about ten or twelve months old.—D. J. ROBETS, M. D., in *Southern Practitioner.*

